# Preparation and *in vivo* evaluation of nanoliposomes containing vancomycin after intravitreal injection in albino rabbits

**DOI:** 10.22038/ijbms.2020.43447.10205

**Published:** 2020-04

**Authors:** Majid Abrishami, Mehrdad Motamed Shariati, Bizhan Malaekeh-Nikouei, Amineh Sadat Tajani, Asma Mahmoudi, Mojtaba Abrishami, Bahman Khameneh

**Affiliations:** 1 Eye Research Center, Mashhad University of Medical Sciences, Mashhad, Iran; 2 Retina Research Center, Mashhad University of Medical Sciences, Mashhad, Iran; 3 Nanotechnology Research Center, Institute of Pharmaceutical Technology, Mashhad University of Medical Sciences, Mashhad, Iran; 4 Department of Pharmaceutical Control, School of Pharmacy, Mashhad University of Medical Sciences, Mashhad, Iran

**Keywords:** Drug delivery, Endophthalmitis, MRSA, Nanopliposomes, Vancomycin

## Abstract

**Objective(s):**

The *in vivo* efficacy of nanoliposomal formulation of vancomycin against methicillin-resistant *Staphylococcus aureus* (MRSA) assessed.

**Materials and Methods:**

Nanoliposomal formulations were prepared and characterized. The *in vivo* study was carried out on rabbits which received liquid culture medium containing MRSA under anesthesia. After 48 hr, the eyes treated with the liposomal and free form of vancomycin. The rabbits were euthanized at predesignate intervals at 12, 24, 48, 96, 144 hr intervals injection. The antibacterial activity of different vancomycin formulations was assayed by the time killing method.

**Results:**

The zeta potential, mean sizes and encapsulation efficacy of liposomal vancomycin were 29.7 mV, 381.93±30.13 nm and 47%, respectively. The results of time–killing studies indicated that the liposomal formula was more effective than the free form of vancomycin.

**Conclusion:**

The results of this study revealed that liposomal vancomycin formulation is a powerful nano-antibacterial agent to combat infectious endophthalmitis.

## Introduction

Infectious endophthalmitis is severe inflammation and sight threating condition that is most commonly caused by an infectious agent involving intraocular fluids and anterior and posterior segments of the eye. The microorganisms are usually Gram-positive bacteria (1, 2). The emergence of multidrug-resistant bacteria is a serious challenge in the management of various complications such as endophthalmitis. Administration of intravitreal antibiotics remains standard treatment in infectious endophthalmitis and vancomycin is a good candidate for this proposes due to coverage of the Gram-positive microorganism (3). This route of administration allows achieving higher drug levels in the vitreous in comparison with topical or systemic administration and avoiding systemic adverse effects (4). Besides these advantages, because of the half-life of the drug and rapid clearance from the vitreous humor, it needs frequent intravitreal injections to reach and to maintain effective therapy. Unfortunately, repeated administrations increase the risk of endophthalmitis, retinal detachment and also lens damages. Additionally, some drugs show local toxicities at their effective dose which lead to unwanted side effects and possible retinal lesions. Consequently, finding the solution for enhancing drug maintenance is desirable (5). In this scenario, on one hand, nanotechnology has been revealed as a promising approach for antibiotics delivery and on another hand, nano-carrier systems can interact with the ocular mucosa and therefore increasing the retention time of the associated drug onto the eye after topical administration (6, 7). Additionally, a wide variety of drugs, including large biomacromolecules or labile drugs can be delivered by these systems and consequently their bioavailability was increased significantly (8). Taken together, employing nano-carriers represents a strategy to overcome problems associated with infectious endophthalmitis. Ocular drug delivery by lipid-based formulations have been introduced for targeted delivery and result in improving drug delivery efficacy (9-12). The bio-distribution of the nano-carriers in the ocular tissue and also their therapeutic advantages have been studied extensively (13, 14). Among these nano-carriers, nanoliposomes as initially most investigated delivery systems have been evaluated for enhancing the corneal penetration of drugs. These carriers represent promising properties such as adhering to the mucosal barrier, prolonging the residence time on the active site and also reducing mucociliary clearance which make them suitable for corneal antibiotic therapy. To date, various studies have been carried out to assay the transport of drugs across the cornea by these carries (15-19).

These carrier systems can increase the residence time of drugs in eye segments and also reduce the toxicity of them. *In vivo* studies indicated that by encapsulation of poorly-stable drugs in liposomes, they can protect from degradation. Moreover, the studies have been conducted to evaluate the residence time of encapsulated drugs in ocular tissues and fluids following intravitreal injection and also their fate (20-23). 

**Figure 1:**
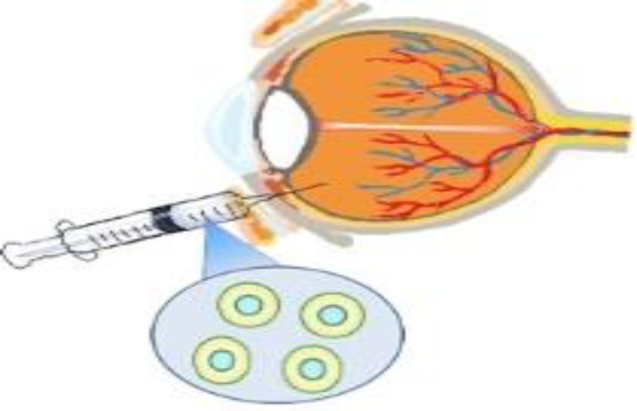
Schematic of the study design

In the present study, the nanoliposomal formulation of vancomycin was prepared and the antibacterial activity of this formulation was compared with the free form of the drug after intravitreal administration against Methicillin-Resistant *Staphylococcus aureus* (MRSA). The schematic of the study design is presented in Figure 1.

## Materials and Methods

***Animal experiment***

New Zealand white rabbits weighing 1.7-2.5 kg were obtained from Pasteur Institute (Tehran, Iran). The sample size was chosen based on that it is a pivotal study and 3 rabbits were assigned for each group. The animals were housed in room lighting with a 12 hr light-dark cycle. The protocol of the study was approved by the regional ethics committee. The research project treated in accommodation with the ARVO assertion for the Use of Animals in Ophthalmic and Vision Research. 

***Materials***

Dipalmitoylphosphatidylcholine (DPPC), stearylamine (SA) and dicetylphosphate (DCP) were ordered from Avanti Polar Lipids (Alabaster, AL). Cholesterol was purchased from Sigma. Vancomycin was obtained from Hakim Pharmaceutical Company (Iran). Chloroform, methanol and Trypticase Soy Agar (TSA) were provided by Merck (Germany).

***Liposome preparation and characterization***

The solvent evaporation method was used for the preparation of vancomycin liposomal formulation. HSPC (hydrogenated soy pc), SA, Chol in a molar fraction of 0.5:1:0.5 were dissolved in chloroform: methanol (2:1) and rotary evaporator (Heidolph, Germany) was used to form a thin lipid film in a round-bottom flask. Vancomycin solution (10 mg/ml) in phosphate-buffered saline (PBS) was then added (phospholipid concentration= 32 μmole/ml) as the aqueous phase. The liposomal suspension was extruded repeatedly, at least 11 times, through 1000, 400 and 100 nm polycarbonate membranes at 45 °C by thermobarrel extruder (Northernlipids, Canada) to produce uniform sized nano-liposome. Non-entrapped vancomycin was separated by dialysis against PBS for 24 hr. Final prepared liposomal formulation stored at 4 °C under an inert atmosphere.

The mean particle sizes, polydispersity index (PDI) and zeta potential of liposomes were assayed by the DLS method (ZetaSizer Nano-ZS; Malvern Instruments, UK). All measurements were performed in triplicate (24).

***Drug encapsulation efficacy***

HPLC-UV/Vis method was used for the determination of 0.25–40 µg/ml concentration range of vancomycin at 229 nm. Samples were chromatographed through a 4.6 mm × 250 mm reverse phase C18 column (Knauer, Germany). The mobile phase was consisted of KH2PO4 (2.5 mM, pH = 2.9): acetonitrile (90:10 v/v %) and flow rate was set on 1.0 ml/min. The volume of each injection was 20 µl. Standard solutions were prepared in the mobile phase before injection (25).

***In vivo*** ***studies***

The animal experiments in the present study conformed to the Animal Research: Reporting of *In Vivo* Experiments (ARRIVE) guidelines (http://www.nc3rs.org.uk/arrive-guidelines) and were approved by the regional ethics committee.

Rabbits were obtained from in house breeding and housed in the institutional animal facilities. They were anesthetized with an intramuscular injection of ketamine hydrochloride (35 mg/kg) and xylazine hydrochloride (5 mg/kg) (26). To produce the endophthalmitis model, 0.1 ml liquid culture medium containing 1000 CFU/mL of MRSA (ATCC 43300) was injected into the vitreous of both eyes of 15 Albino rabbits by an insulin injector with a 25 gauge needle, 2 mm posterior to the limbus in the superior-temporal quadrant. After 48 hr, eyes were examined and all of them show the clinical signs of endophthalmitis including red reflex disturbance, anterior chamber, and vitreous inflammation. Liposomal vancomycin (400 µg/0.1 ml) was injected intravitreally in the right eyes and free formulation of vancomycin (400 µg/0.1 ml) was injected in the left eyes. Samples were obtained via 5 mm sclerotomies 4 mm posterior to the limbus sample and vitreous were evacuated from the eyes using an insulin syringe. Vitreous sampling of both eyes was done after 12, 24, 48, 96, 144 hr intervals from the antibiotic injection. Euthanasia was performed with intraperitoneal pentobarbitone (60–150 mg/kg).

***Time–killing studies***

The bactericidal activity of different formulations of vancomycin against bacteria was also evaluated using time-kill curves. These curves were performed using the liposomal and free form of vancomycin at equal concentrations. The number of viable cells was determined by performing 10-fold serial dilutions of these suspensions and plating 50 µl of the dilutions in triplicate on TSA plates. The plates were incubated at 37 ^°^C for 18–24 hr.

***Statistical analysis***

Statistical testing was performed using SPSS for Windows software (version 16, SPSS, Inc.). Variables are expressed as Mean±SD. Analysis of variance (ANOVA) was used to evaluate the significance of differences among groups. Independent T-test was used to evaluate statistical significance in the difference between the means of the two groups. The *P-values* of less than 0.05 were considered to be statistically significant.

## Results

***Liposomal characterization***

The liposomal properties including Z-average, PDI, zeta potential and encapsulation efficiency were illustrated in Tables 1 and 2. The zeta potential of liposomal formulations was approximately 29.7 mV due to the presence of stearylamine in the lipid phase. According to the results, the encapsulation efficacy of liposomal vancomycin was approximately 47%.

***In vivo*** ***studies***

**Figure 2:**
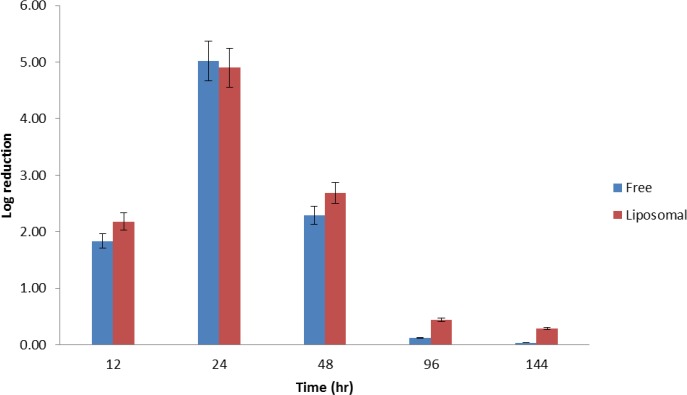
Time-kill curves of each antibacterial formulation against MRSA (ATCC 43300). Logarithmic reduction of bacterial viability after 12, 24, 48, 96 and 144 hr of exposures to the different formula. Points represent means and error bars (n=3)

Figure 2 showed that the liposomal formulation was significantly more effective than the free form of vancomycin at each tested time interval (*P*<0.05). It should be pointed out that the percentage of mean reduction of bacteria by liposomal formulation was significantly more pronounced over studied time (*P* <0.05). 

**Table 1:** Z-average, polydispersity index (PDI) and zeta potential of liposomal formulation (mean±SD, n=3

Sample	Size	PDI	Zeta Potential (mV)
Vancomycin liposomal formulation	381.93 ± 30.13	0.496 ± 0.0488	29.7 ± 4.7

**Table 2:** Encapsulation efficacy of liposomal formulation (Mean±SD, n=3)

Sample	Encapsulation efficacy (%)	Drug-loaded concentration (mg/ml)
Vancomycin liposomal formulation	47%	4.7 mg/ml

## Discussion

The present study aimed to evaluate the efficacy of nanoliposomal formulation of vancomycin after intravitreal injection against MRSA induced endophthalmitis in an experimental model. According to the results (Tables 1 and 2), the zeta potential and encapsulation efficacy of liposomal formulations were approximately 29.7 mV and 47%, respectively. These data were in line with previously published data and indicated that hydrophilic drugs such as vancomycin showed lower encapsulation efficiency for the hydrophobic ones (27, 28). Additionally, the positive charge of particles can help in better interaction of the particulate system with mucosal and ocular surfaces (29, 30). 

As shown in Figure 1, based on the bacteria counting assay, the liposomal formulation was significantly more effective than the free form of vancomycin at each tested time interval (*P*<0.05). Increasing the efficiency of the drug upon encapsulation in liposomes for ocular drug delivery has been demonstrated previously (30, 31). Their results indicated that positively charged and nano-sized liposomes showed enhanced therapeutic effects. As mentioned above, positively charged liposomes can interact better with ocular surfaces than neutral and anionic ones. This ability of liposomes might be due to electrostatic attraction forces between the positively-charged amine groups in liposomal structure and negatively-charged phosphate groups in the mucosal barrier (17, 29). In another study, trimethyl chitosan-coated liposomes of vitamin A palmitate were used for ocular delivery. The results of this study indicated that the liposomal formula showed sustained drug release behavior, prolonged ocular retention and improved corneal penetration (32). Additionally, based on the results, the percentage of mean reduction of bacteria by liposomal formulation was significantly more pronounced over time. These observations were in agreement with a previously published study (17). The data indicated that the formulation system showed enhanced antibacterial activities against both Gram-positive and -negative bacteria and also the carrier system can inhibit the growth of Gram-negative bacteria in rabbits’ eye models. Various mechanisms have been mentioned for enhanced antibacterial activity of particulate systems with respect to the free form of the drug. Enhancing the ocular retention time of drugs is to be a more probable mechanism (17, 32, 33). Consequently, employing positively charged particles for ocular delivery systems seems to be logical. 

## Conclusion

The results of this study revealed that liposomal vancomycin formulation is a powerful nano-antibacterial agent to combat infectious endophthalmitis and has potential merit for further investigations. The results of this study highlighted the advantages of liposomal vancomycin in the treatment of infectious endophthalmitis. The developed liposomal formulation with sufficient drug encapsulation rate showed antibacterial activity *in vivo* against MRSA.
